# 100 Hz Electroacupuncture Alleviated Chronic Itch and GRPR Expression Through Activation of Kappa Opioid Receptors in Spinal Dorsal Horn

**DOI:** 10.3389/fnins.2021.625471

**Published:** 2021-02-16

**Authors:** Hong-Ping Li, Xiao-Yu Wang, Chao Chen, Jing-Jing Li, Chi Yu, Li-Xue Lin, Zi-E Yu, Zhi-Yuan Jin, He Zhu, Hong-Chun Xiang, Xue-Fei Hu, Jie Cao, Xiang-Hong Jing, Man Li

**Affiliations:** ^1^Department of Neurobiology, School of Basic Medicine, Tongji Medical College, Huazhong University of Science and Technology, Wuhan, China; ^2^Institute of Acupuncture and Moxibustion, China Academy of Chinese Medical Sciences, Beijing, China; ^3^Department of Neurology, Tongji Hospital, Tongji Medical College, Huazhong University of Science and Technology, Wuhan, China

**Keywords:** EA, chronic itch, spontaneous scratching, GRPR, DYN-A, kappa opioid receptors

## Abstract

**Background:**

Clinical studies have shown that electroacupuncture (EA) alleviates chronic itch. Gastrin-releasing peptide receptor (GRPR) and dynorphin (DYN) in the spinal dorsal horn positively or negatively regulate itch, respectively. However, which frequency of EA is effective on relieving chronic itch and reducing the expression of GRPR, whether DYN-A in the spinal cord is involved in the underlying mechanism of the antipruritus effect of EA remains unknown.

**Methods:**

The mixture of acetone and diethyl ether (1:1) [designated as AEW (acetone/diethyl ether and water) treatment] was used to induce the dry skin model of chronic itch. EA was applied to Quchi (LI11) and Hegu (LI4). Western blot was used to detect the expression of GRPR and DYN-A. Immunofluorescence was used to detect the expression of DYN-A.

**Results:**

The AEW administration induced remarkable spontaneous scratching, enhanced the expression of GRPR, and reduced the expression of DYN-A. Compared with the sham EA, 2 Hz EA, or 15 Hz EA group, 100 Hz EA was the most effective frequency for relieving chronic itch, reducing the expression of GRPR, and increasing the expression of DYN-A in the cervical dorsal horn. Furthermore, intraperitoneal injection of kappa opioid receptors (KORs) antagonist nor-Binaltorphimine dihydrochloride (nor-BNI) significantly reversed the effect of 100 Hz EA on the inhibition of both itching behavior and GRPR expression.

**Conclusion:**

EA at 100 Hz is the most effective frequency that inhibits chronic itch and GRPR expression through activation of KORs in the spinal dorsal horn, which can effectively guide the clinical treatment and improve the antipruritic effect of acupuncture.

## Introduction

Itch is an aversive sensory associated with an actual disruption to the skin, reminding us of the potential physical threats (Ross, 2011) and an emotional experience that provokes an appetite to scratch. It is a common symptom in patients with atopic dermatitis, xerosis, and systemic disorders containing chronic renal failure and cholestasis (Di Nardo et al., 1998). Although irritants may be removed from the skin by scratching, which can transiently relieve itch at least, the itch-scratch-itch cycle is a characteristic of chronic itch (Weisshaar and Dalgard, 2009). Previous studies have indicated that the mechanisms of chronic itch may involve sensitization of itch-signaling pathways (Simone et al., 1991; Akiyama et al., 2012), so antihistamine medicine does not work well in chronic itch diseases. Lee et al. (2018) found that electroacupuncture (EA) at Hegu (LI4) and Quchi (LI11) acupoints reduced scratching induced by 5’-guanidinonaltrindole [GNTI, a kappa opioid receptor (KOR) antagonist] in mice. Previous clinical and experimental studies have shown that EA can effectively reduce chronic itching caused by psoriasis, atopic dermatitis, urticaria, uremic pruritus, and other chronic allergic skin diseases (Duo, 1987; Che-Yi et al., 2005; Kim et al., 2010; Jung et al., 2014; Wang et al., 2019; Zhang et al., 2020a, b). However, the mechanism of EA to relieve chronic itch is still unclear, which is hindering its clinical application.

Interestingly, gastrin-releasing peptide receptor (GRPR) and dynorphin (DYN) in the spinal dorsal horn positively or negatively regulate itch, respectively. Gastrin-releasing peptide (GRP) is a neurotransmitter in mammalian cells/neurons and plays a role through combining with its specific receptor—GRPR, a G-protein-coupled receptor (GPCR) (Weber, 2015), which lay in the superficial layer of the spinal dorsal horn (Sun and Chen, 2007). Previous studies showed that signaling pathway in the central nervous system mediated by GRPR is important in many physiological processes (Liu et al., 2014; Takanami and Sakamoto, 2014; Kiguchi et al., 2016). According to previous reports in the spinal cord, the GRP/GRPR system participates in the regulation of itch-specific transmission rather than the transmission of pain. Ablation of GRPR neurons in the spinal cord caused specific reduction in itch, but not pain (Sun and Chen, 2007). It has been reported that itch and pain may transfer information through different neural pathways (Ji, 2015) and the neural pathway of itch in the spinal cord includes the GRPR neurons (Pereira et al., 2015). Studies have shown that the higher the GRPR expression in the spinal dorsal horn, the greater the degree of itch (Nattkemper et al., 2013). However, it is not clear whether EA can inhibit the expression of GRPR, thus desensitizing the itch-signaling pathways and relieving chronic itch.

Itch, as a unique sensation like pain, has an interaction with pain (Lagerstrom et al., 2010). It is not a surprise that the pain associated with molecules, such as endogenous opioid system, could modulate scratching behavior (Cowan et al., 2015; Ko, 2015). A previous study demonstrated that mice intrathecally injected with morphine, a mu opioid receptor (MOR) agonist, induced dose-dependent scratching behavior (Liu et al., 2011). Conversely, KOR agonist nalfurafine could reduce both histamine-dependent and histamine-independent scratching behavior in mice (Inan and Cowan, 2004; Wang et al., 2005). Moreover, KORs agonist nalfurafine can inhibit spontaneous scratching (Inan and Cowan, 2006). A further study implicated that B5-I^+^ neurons in the spinal cord expressed the kappa opioid endogenous ligand DYN, which could activate the KOR in GRPR^+^ neurons and inhibit itch transmission elicited by various pruritogens (Kardon et al., 2014). It is suggested that KOR, but not MOR, may participate in alleviation of itch.

It has been found that EA can increase the expression of DYN (Han, 2003) in the lumbar enlargement of rats with atopic dermatitis (Jung et al., 2014). In addition, high-frequency (100 Hz) EA increased the content of DYN in perfusate from the spinal cord of rats (Sun and Han, 1989) and the level of DYN-A in the plasma of heroin addicts by radioimmunoassay (Mu et al., 2010), which indicated that 100 Hz EA relieving itch may be related to DYN-A. In contrast, 2 Hz EA increased the release of endogenous ligands of MORs, whereas 15 Hz EA produced a partial activation of both MOR and KOR (Guo et al., 1997; Ulett et al., 1998; Han, 2004). It indicated that 100 Hz EA should have better antipruritic effect than 2 and 15 Hz EA. However, which frequency of EA is most effective on relieving chronic itch and reducing the expression of GRPR, and whether DYN-A in the spinal cord is involved in the underlying mechanism of the antipruritic effect of EA remains unknown.

Here, we firstly compared the effect of different frequencies of EA on spontaneous scratching behavior, GRPR, and DYN-A expression in the cervical cord, in order to screen out the most effective frequency of EA. In addition, we investigated whether EA at the effective frequency inhibits chronic itch and GRPR expression through promoting the release of DYN-A and activating KOR in the spinal cord of mice with chronic itch.

## Materials and Methods

### Animal Grouping

All experiments were carried out on adult C57BL/6 mice (male, 18–20 g) purchased from Beijing Vital River Laboratory Animal Technology Co., Ltd. The study protocol was approved by the Animal Care Committee at Huazhong University of Science and Technology. The mice were individually housed in cages with a 12-h light–dark cycle (light from 7:00 a.m. to 7:00 p.m.) with *ad libitum* access to water and food.

**FIGURE 1:**
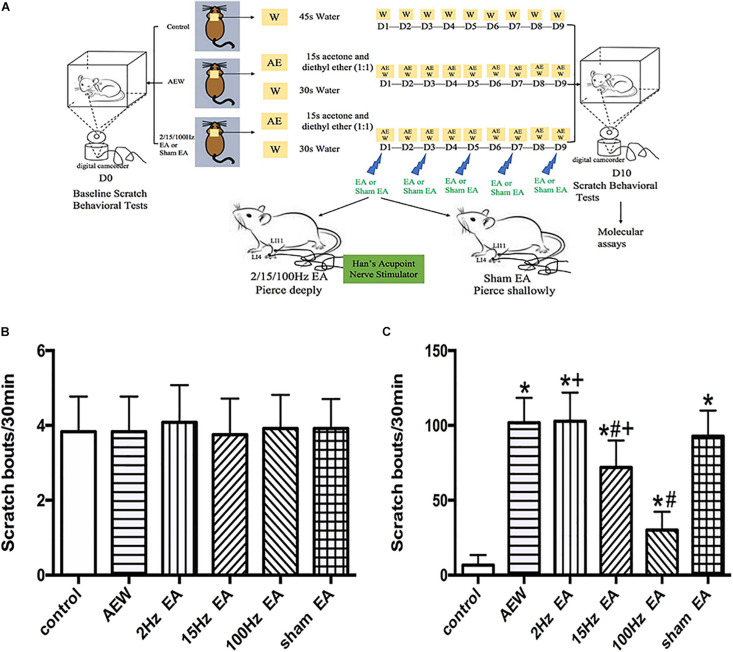
Time course of the effect of EA on the mixture of acetone and diethyl ether induced scratch bouts. **(A)** An experiment design time line with the day and time of all manipulations. **(B)** The baseline scratch bouts in control, AEW, 2 Hz EA, 15 Hz EA, 100 Hz EA, and sham EA mice. **(C)** The scratch bouts in control and AEW mice and the effects of 2 Hz EA, 15 Hz EA, 100 Hz EA, and sham EA on scratch bouts in AEW mice. EA was administered for 30 min, once every other day for 9 days, starting from 1 day after AEW administration. Data are expressed as mean ± SEM (*n* = 12 mice in each group). *P < 0.05, compared with the control group; ^#^*P* < 0.05, compared with the sham EA group; +*P* < 0.05, compared with the 100 Hz EA group.

Two separate experiments were conducted. At first, 72 mice were randomly divided into the following six groups with SPSS, version 26.0: control (control of chronic itch), AEW (chronic itch), 2 Hz EA (chronic itch + 2 Hz EA), 15 Hz EA (chronic itch + 15 Hz EA), 100 Hz EA (chronic itch + 100 Hz EA), and sham EA (chronic itch + sham EA) groups. Secondly, 60 mice were randomly divided into five groups: control, AEW, EA + NS (chronic itch + 100 Hz EA + normal saline), EA + nor-BNI (chronic itch + 100 Hz EA + nor-BNI), and sham EA groups. The observer was not aware of the group allocation during the experiment; the experiment operator was not aware of the group allocation during the conduct of the experiment; the effect evaluator was not aware of the group allocation during the outcome assessment and the data analysis. An experiment design time line with the day and time of all manipulations is presented in Figures 1, 6. At the end of the 10th day, a humanitarian final point was made as followed. The mice were euthanized with intraperitoneal injection (i.p.) of sodium pentobarbital (250 mg/kg).

### Animal Models

AEW treatments reduced stratum corneum hydration, enhanced transepidermal water loss, and inhibited spontaneous scratching. Naloxone and naltrexone (opioid antagonists) inhibited spontaneous scratching in AEW-treated mice. It is suggested that spontaneous scratching of AEW-treated mice is an itch-related response and a useful model for studying the mechanisms of dry skin pruritus (Miyamoto et al., 2002; Akiyama et al., 2010a, b). In order to induce chronic itch of the dry skin model in mice, we followed the protocol reported in a previous study (Akiyama et al., 2012). On the test day, a mixture of acetone and diethyl ether (1:1) was applied to the shaved area by a piece of wet gauze (the area is approximately 15 × 15 mm^2^) at the neck for 15 s and then using another gauze infiltrated by distilled water for another 30 s, twice a day for 9 days consecutively, which was designated as AEW (acetone/diethyl ether and water) treatment. Mice in the control group were identically treated with distilled water for 45 s (designated as control treatment).

### EA Treatment

The animals in different groups were habituated to the restricting bag for 3 days before EA treatment, 30 min each day, to avoid stress. In the EA administration group, the mice received EA treatment on the left “Hegu” (LI4) and “Quchi” (LI11) starting from 1 day after the first AEW treatment in the restricting bag, once every other day for 5 times (Figure 1A). The acupoint “Hegu” (LI4) is located on the dorsum of the hand, radial to the midpoint of the forepaw’s second metacarpal bone (Yen et al., 2019), and “Quchi” (LI11) is located at the end of the lateral transverse elbow crease (Liu et al., 2016). The selection of acupoints is based on previous studies and our preclinical studies (Che-Yi et al., 2005; Ni et al., 2018). EA (1 mA) was administered with different frequencies (2, 15, or 100 Hz) for 30 min. Two acupuncture needles (0.25 mm outer diameter) were inserted 2–3 mm into two acupoints on the left side corresponding to the LI4 and LI11 for 30 min (Yim et al., 2007). The current was transferred by a modified current constant Han’s Acupoint Nerve Stimulator (LH202; Huawei Co., Ltd., Beijing, China). We observed the shaking of the left forelimb muscle as the criterion for success of the EA, and the successful rate is 100%. For the sham EA group, acupuncture needles were only shallowly inserted into the LI4 and LI11 for 30 min without electrical stimulation.

### Drug Administration

nor-Binaltorphimine dihydrochloride (nor-BNI; Sigma, St. Louis, MO, United States) was dissolved in sterile 0.9% NaCl (normal saline, NS) at a concentration of 2.5 mg/ml. Each mouse in the nor-BNI treatment group was intraperitoneally injected with 20 mg/kg 18–20 h before EA, once every other day for 5 times. The EA + NS group accepted an equal volume of vehicle (NS) injection.

### Scratch Behavioral Tests

To score scratching, mice were placed into individual observation Plexiglas recording arena with a transparent cover described previously (Nojima et al., 2003). We used a digital camcorder to videotape the mice’s behavior for 30 min (Figure 1A). The number of scratch bouts was counted. A series of one or more scratching movements by the hindlimb directed toward the treatment area was regarded as one scratching bout, which ended when the mouse either bit/licked the toes or placed the hindlimb on the floor. AEW or control treatments were performed twice daily (09:00 a.m. and 17:00 p.m.) for 9 consecutive days. Then, 16–20 h after the second AEW administration on the 9th day, spontaneous behavior was videotaped for 30 min on the 10th day. Animal behavior in different groups was measured randomly to reduce potential confounders among groups.

### Western Blot

After taking weight and observing general health, mice were anesthetized with sodium pentobarbital (250 mg/kg, i.p.), euthanized, and sacrificed by cervical dislocation. This was followed by excision of the cervical cord tissues (C1–C7). The cervical cord tissues (C1–C7) of mice were removed and homogenized in RIPA lysis buffer with 50 mg/ml (Beyotime Biotechnology, Nanjing, China). The homogenates were centrifuged at 12,000 × *g* for 15 min at 4°C. The concentrations of protein obtained from the supernatant were detected by the Enhanced BCA Protein Assay Kit (Beyotime Biotechnology, China).

Twenty mg protein of each tissue was denatured at 95°C for 5 min. And then, 40 μg proteins were separated on an 8–10% glycine–SDS-PAGE gel and transferred onto a polyvinylidene fluoride membrane. Then, the transferred blots were blocked in 5% non-fat dry milk in Tris-buffered saline (TBS). The membrane was incubated with rabbit anti-Dynorphin A antibody (1:1,000; Abcam, Hong Kong), rabbit anti-GRPR antibody (0.75 μg/ml; Cloud-Clone Corp), and horseradish peroxidase (HRP)-conjugated alpha Tubulin Mouse Monoclonal antibody (1:10,000; Proteintech) at 4°C overnight. The membranes were incubated with HRP-conjugated goat anti-rabbit secondary antibody (Santa Cruz Biotechnology) at 1:20,000. The enhanced chemiluminescence method (ECL Plus Western blot detection reagents; Pierce, Rockford, IL, United States) was applied to show the protein bands basing on the manufacturer’s protocol. X-ray films were scanned, and the intensities of corresponding bands (55 kDa for tubulin, 43 kDa for GRPR, and 28 kDa for DYN-A) were measured using Kodak 1D. The optical density of each band was measured with NIH ImageJ software (Bethesda, MD, United States) and normalized with the tubulin, a housekeeping gene. All of the experimental results were shown as the relative change over the protein amount in the control group.

### Histology and Immunofluorescence Labeling

After taking weight and observing general health, mice were anesthetized with sodium pentobarbital (250 mg/kg, i.p.), euthanized, and sacrificed by perfusing through the ascending aorta with 37°C normal saline, followed by 4% paraformaldehyde dissolved in 0.1 M phosphate-buffer solution (PBS, pH 7.4, 4°C). After the perfusion, the inflammatory skin tissue in the neck and the C1–C7 segments of the cervical cord were immediately removed, post-fixed, and cryoprotected at 4°C for hematoxylin–eosin (H&E) and immunofluorescence labeling, respectively.

For H&E staining, the post-fixed skin tissue was embedded in paraffin and was cut into 4 μm thickness sections. The sections were mounted on glass slides and then stained with H&E used for evaluation of epidermal thickness.

For immunofluorescence labeling, the sections from the cervical cord were cut on a cryostat at 20 μm thickness. And then, the sections were processed for immunofluorescence labeling as described previously (Wu et al., 2016). Briefly, the sections were blocked with 5% donkey serum and then incubated with primary antibodies overnight at 4°C: rabbit anti-Dynorphin A antibody (1:200, Abcam). Then, the sections were incubated with secondary antibodies corresponding with primary antibodies from Jackson ImmunoResearch (West Grove, PA, United States): donkey anti-rabbit IgG conjugated with Dylight 594 (1:400).

An Olympus BX51 fluorescence microscope was used to view the sections, and images were obtained using a Qimaging Camera and QCapture software as described before (Yuan et al., 2017). A total of 5–6 sections from the skin or cervical cord were randomly selected in each mouse. Anatomic outlines of the epidermis of the neck skin or the gray matter of the cervical cord were plotted, and epidermal thickness or the area of DYN-A positive immunoreactivity was measured by using ImageJ software.

### Statistical Analysis

All data were expressed as the mean ± standard error of the mean (SEM). Normality of data was checked by Shapiro–Wilk test. All statistical (behavioral data and biochemical data) comparisons were performed with one-way ANOVA and Bonferroni for separation of means (SPSS, version 26.0) to determine the statistical difference between individual groups. The criterion for statistical significance was set at *P* < 0.05.

## Results

### Effect of EA on the Itch Behavior and Histological Features of Chronic Itch Model Mice

An experiment design time line with the day and time of all manipulations is presented in Figure 1A. The baseline scratch bouts in all the experimental groups before AEW treatment were similar (Figure 1B). The scratch bouts significantly increased 9 days after AEW administration compared with the control group (Figure 1C). EA at 15 or 100 Hz, but not 2 Hz, significantly decreased the scratch bouts compared with the sham EA group (*P* < 0.05, Figure 1C). The scratch bouts in the 2 Hz EA or 15 Hz EA group were significantly higher than that in the 100 Hz EA group (*P* < 0.05, Figure 1C). What we screened the effective frequency of EA to relieve itch was 100 Hz.

**FIGURE 2:**
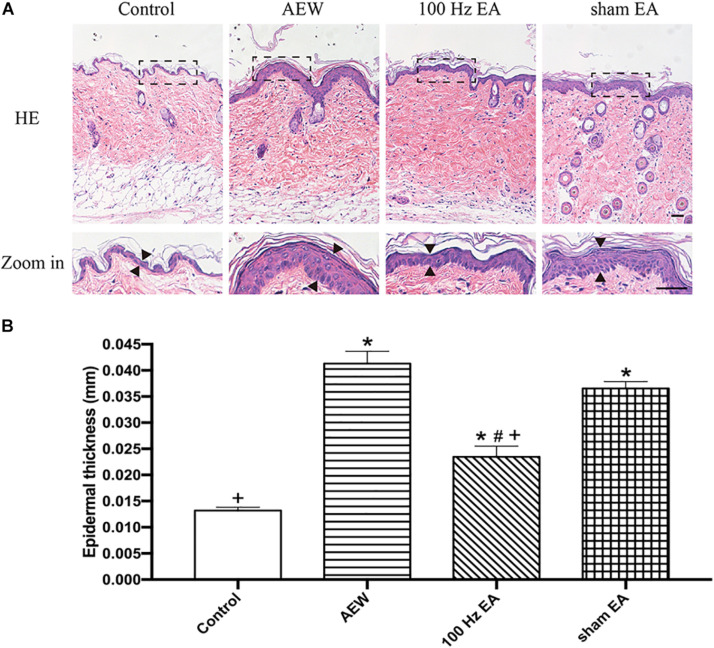
Effect of EA on the morphological and histological features in inflammatory neck skin. **(A)** Representative macroscopic views and H&E staining of cross-sectional slices of the dorsal skin in the control, AEW, 100 Hz EA, and sham EA groups. Scale bar, 50 μm. **(B)** Summary data show the epidermal thickness of the neck skin of four groups. Data are expressed as mean ± SEM (*n* = 6 mice in each group). *P < 0.05, compared with the control group; ^#^*P* < 0.05, compared with the sham EA group; ^+^*P* < 0.05, compared with the AEW group.

In order to determine whether EA affects the immune response (inflammation) of the AEW model or affects the itching sensation pathway, we used H&E staining to investigate the pathological changes of the skin in the chronic itch model. H&E staining was performed on the inflammatory neck skin of the mice on day 10 after AEW application. It revealed significant epidermal hyperplasia, significantly increased telangiectasia, and inflammatory cell infiltration in the AEW, EA, and sham EA groups. EA significantly improved the histologic changes caused by AEW. The skin thickness of the AEW group was significantly increased compared with the control group, which was decreased in the EA group (*P* < 0.05, Figure 2).

### Effect of EA at Different Frequencies on the Expression of GRPR Protein in the Cervical Cord

**FIGURE 3:**
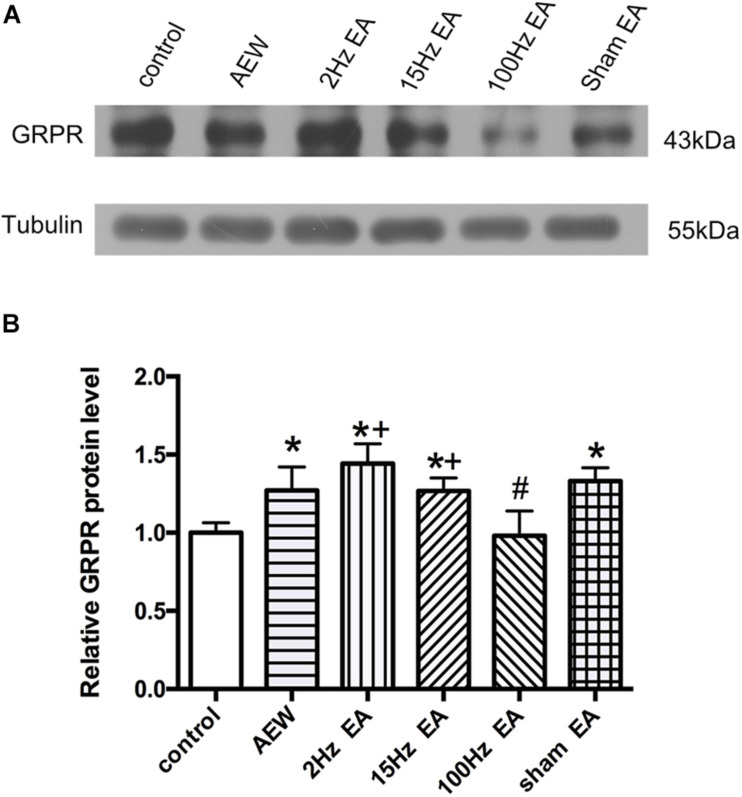
Effect of EA at different frequencies on the expression of GRPR protein in the cervical cord. **(A)** Representative gel image shows the protein level of GRPR in the control, AEW, 2 Hz EA, 15 Hz EA, 100 Hz EA, and sham EA groups. **(B)** Summary data show the percentage change in GRPR protein level in six groups. Tubulin (55 kDa) was used as a loading control. The protein band at 43 kDa corresponds to the GRPR protein. Data are expressed as mean ± SEM (*n* = 6 mice in each group). *P < 0.05, compared with the control group; ^#^*P* < 0.05, compared with the sham EA group; ^+^*P* < 0.05, compared with the 100 Hz EA group.

It has been confirmed that GRPR is a specific receptor of itch (Sun and Chen, 2007). We used western blot to investigate the effect of EA on the expression of GRPR in the cervical cord. On the 9th day, the protein level of GRPR in the cervical cord of AEW mice was significantly higher than that of the control group (*P* < 0.05, Figures 3A,B). Compared with the sham EA group, EA at 100 Hz, but not 2 or 15 Hz, significantly decreased the protein level of GRPR in the cervical cord. The expression of GRPR in the 2 and 15 Hz EA groups was significantly higher than that in the 100 Hz EA group (*P* < 0.05, Figures 3A,B). This result suggested that 100 Hz EA suppressed the expression of GRPR in the cervical cord to relieve itch.

### Effect of EA at Different Frequencies on the Expression of DYN-A Protein in the Cervical Cord

**FIGURE 4:**
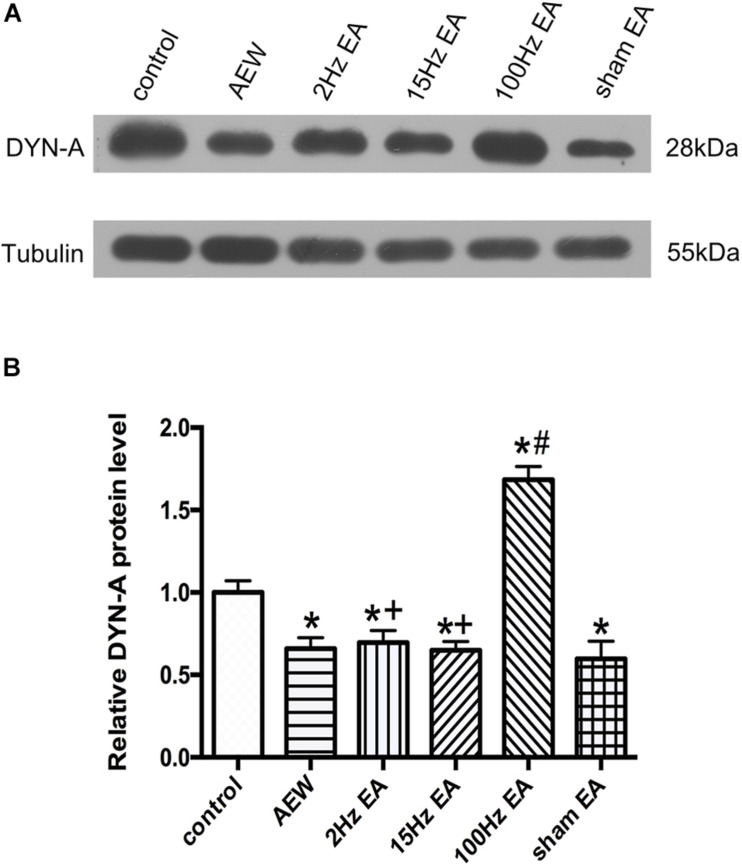
Effect of EA at different frequencies on the expression of DYN-A protein in the cervical cord. **(A)** Representative gel image shows the protein level of DYN-A in the control, AEW, 2 Hz EA, 15 Hz EA, 100 Hz EA, and the sham EA groups. **(B)** Summary data show the percentage change in DYN-A protein level in six groups. Tubulin (55 kDa) was used as a loading control. The protein band at 28 kDa corresponds to the DYN-A protein. Data are expressed as mean ± SEM (*n* = 6 mice in each group). *P < 0.05, compared with the control group; ^#^*P* < 0.05, compared with the sham EA group; ^+^*P* < 0.05, compared with the 100 Hz EA group.

Previous study has shown that EA can increase the expression of DYN in the L4–L6 of rats with atopic dermatitis (Jung et al., 2014). We used western blot to investigate the effect of EA on the expression of DYN-A in the cervical cord. On the 9th day, the protein level of DYN-A in the cervical cord of AEW mice was significantly lower than that of the control group (*P* < 0.05, Figures 4A,B). Compared with the sham EA group, EA at 100 Hz, but not 2 or 15 Hz, significantly increased the protein level of DYN-A in the cervical cord (*P* < 0.05, Figures 4A,B). The protein level of DYN-A in the 2 or 15 Hz EA group was significantly lower than that in the 100 Hz EA group (*P* < 0.05, Figures 4A,B). These results suggested that 100 Hz EA up-regulated the protein expression of DYN-A in the cervical cord to relieve itch.

**FIGURE 5:**
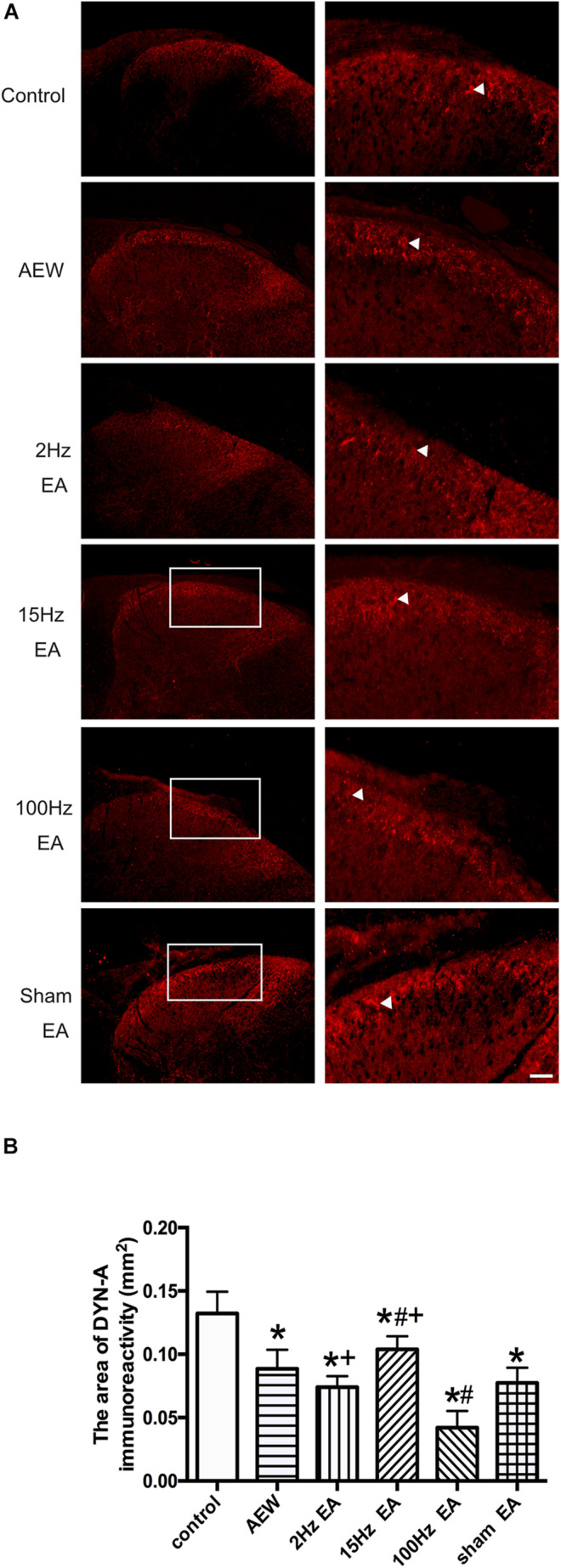
Effect of EA at different frequencies on the area of DYN-A immunoreactivity in the cervical cord. **(A)** Representative images showing DYN-A immunoreactivity in the cervical dorsal horn of the control, AEW, 2 Hz EA, 15 Hz EA, 100 Hz EA, and sham EA groups (left panels) and corresponding enlarged images (right panels). Arrows represent DYN-A positive neurons. Scale bar, 100 μm. **(B)** Summary data show the area of DYN-A immunoreactivity in different groups. Data are expressed as mean ± SEM (*n* = 7 mice in each group). *P < 0.05, compared with the control group; ^#^*P* < 0.05, compared with the sham EA group; ^+^*P* < 0.05, compared with the 100 Hz EA group.

In addition, EA increased the release of DYN in perfusate from the spinal cord of rats (Sun and Han, 1989) and the plasma of heroin addicts (Mu et al., 2010) by radioimmunoassay. Using immunofluorescence, we found that DYN-A was expressed in the superficial layer of the cervical dorsal horn in each group (Figure 5A). On the 9th day, the area of DYN-A positive immunoreactivity in the AEW group significantly decreased compared with the control group (*P* < 0.05, Figures 5A,B). Compared with the sham EA group, the area of DYN-A immunoreactivity in the cervical dorsal horn significantly increased in the 15 Hz EA group and markedly decreased in the 100 Hz EA group. The area of DYN-A immunoreactivity in the 2 or 15 Hz EA group significantly increased compared with the 100 Hz EA group (*P* < 0.05, Figures 5A,B). These results suggested that 100 Hz EA promoted the release of DYN-A in the cervical dorsal horn to relieve itch.

### EA at 100 Hz Down-Regulated Itch Behavior Through KORs

**FIGURE 6:**
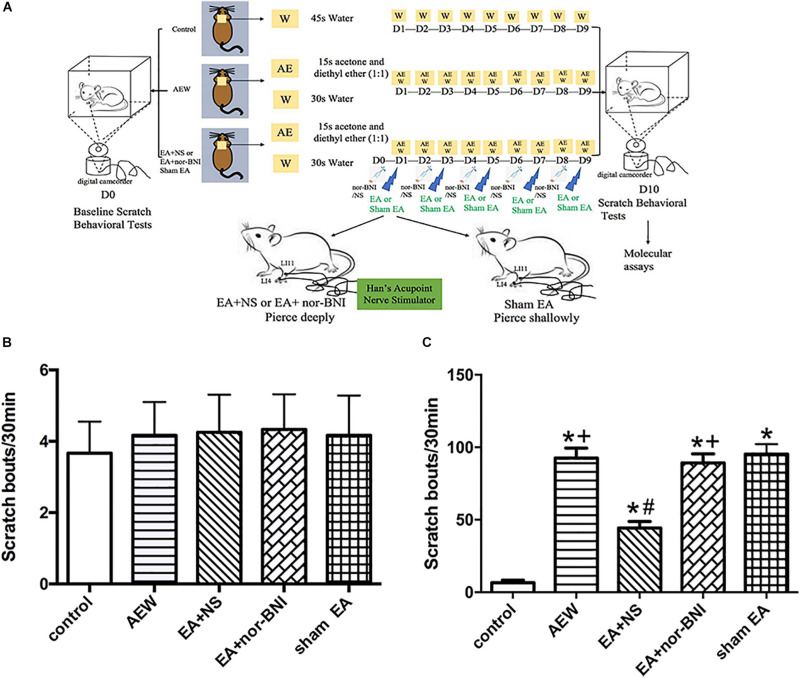
Time course of the effects of kappa opioid receptors on the mixture of acetone and diethyl ether induced scratch bouts. **(A)** An experiment design time line with the day and time of all manipulations. **(B)** The baseline scratch bouts in the control, AEW, EA + NS, EA + nor-BNI, and sham EA groups. **(C)** The scratch bouts in control and AEW mice and the effects of kappa opioid receptors on scratch bouts in AEW mice. EA at 100 Hz was administered for 30 min, once every other day for 9 days, starting from 1 day after AEW administration. Data are expressed as mean ± SEM (*n* = 12 mice in each group). *P < 0.05, compared with the control group; ^#^*P* < 0.05, compared with the sham EA group. ^+^*P* < 0.05, compared with the EA + NS group.

An experiment design time line with the day and time of all manipulations is presented in Figure 6A. The baseline scratch bouts in all the experimental groups before AEW treatment were similar (Figure 6B). The scratch bouts in AEW significantly increased 9 days after AEW administration compared with the control group (*P* < 0.05, Figure 6C). EA at 100 Hz significantly decreased the scratch bouts compared with the sham EA group (*P* < 0.05, Figure 6C). I.p. of nor-BNI, but not normal saline, significantly reversed the effect of 100 Hz EA on relieving itch (*P* < 0.05, Figure 6C). The results showed that 100 Hz EA relieves itch through KOR.

### EA at 100 Hz Down-Regulated the Expression of GRPR in the Cervical Cord Through KORs

**FIGURE 7:**
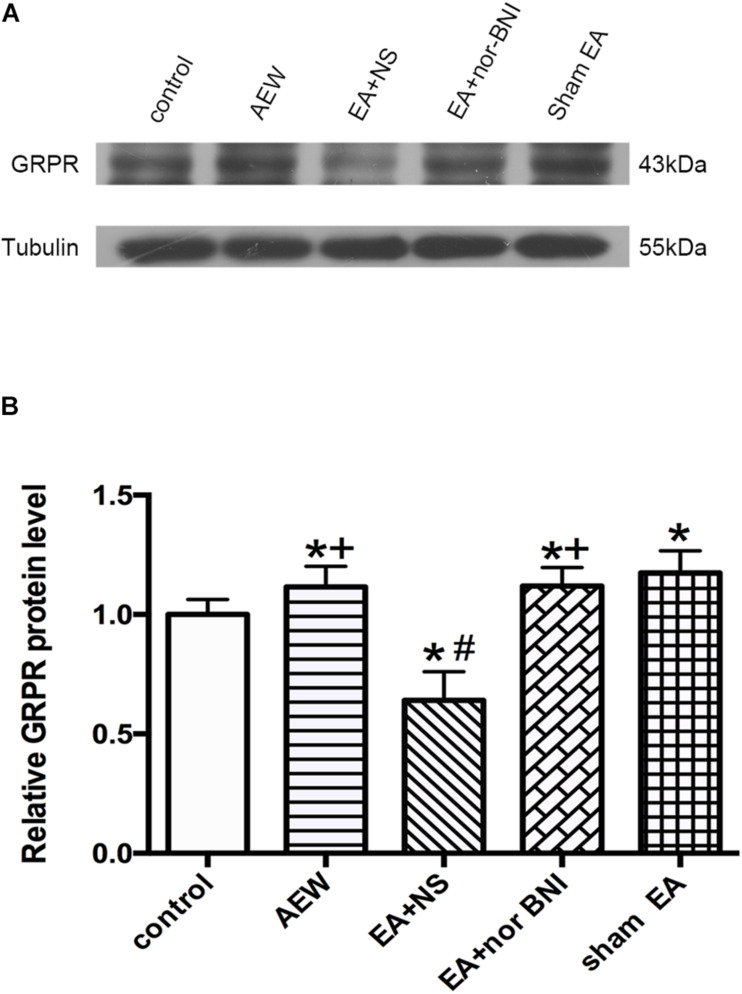
Effect of KOR on the expression of GRPR protein in the cervical cord. **(A)** Representative gel image shows the protein level of GRPR in the control, AEW, EA + NS, EA + nor-BNI, and sham EA groups. **(B)** Summary data show the percentage change in GRPR protein level in five groups. Tubulin (55 kDa) was used as a loading control. The protein band at 43 kDa corresponds to the GRPR protein. Data are expressed as mean ± SEM (*n* = 6 mice in each group). *P < 0.05, compared with the control group; ^#^*P* < 0.05, compared with the sham EA group. ^+^*P* < 0.05, compared with the EA + NS group.

We used western blot to investigate the effect of KOR on the expression of GRPR in the cervical cord. On the 9th day, the protein level of GRPR in the cervical cord of AEW mice was significantly higher than that of the control group (*P* < 0.05, Figures 7A,B). Compared with the sham EA group, the EA + NS group significantly decreased the protein level of GRPR in the cervical cord. I.p. of nor-BNI significantly increased the protein level of GRPR compared with the EA + NS group (*P* < 0.05, Figures 7A,B).

## Discussion

Previous clinical studies have shown that EA can remain an effective way of suppressing chronic itch, including many diseases, such as seasonal allergic rhinitis (Xue et al., 2015), atopic dermatitis (Tan et al., 2015), dermatology (Ma and Sivamani, 2015), and so on. However, it has not been illustrating which frequency is the most effective, neither the underlying mechanisms. For the first time, we found that compared with the sham EA, 2 Hz EA, or 15 Hz EA group, 100 Hz EA is the most effective frequency on relieving chronic itch, reducing the expression of GRPR, and increasing the expression of DYN-A in the cervical dorsal horn. Furthermore, i.p. of KORs antagonist nor-BNI significantly reversed the effect of 100 Hz EA on the inhibition of both itching behavior and GRPR expression.

It has been shown that GRPR is crucial for itch signal transmission at the spinal level and a specific receptor that contributes to mediate the itch in the spinal dorsal horn (Sun and Chen, 2007). Consistently, ablation of GRPR neurons in the spinal cord caused specific reduction in itch, but not pain (Sun and Chen, 2007). In this study, we found that EA at 100 Hz, but not 2 or 15 Hz, decreased the expression of GRPR in the cervical cord. The inhibitory effect of EA on GRPR expression parallels with its antipruritic effect, which is consistent with the relation of GRPR expression and chronic itch (Nattkemper et al., 2013). Previous studies have found that GRPR is expressed only in the neurons in the dorsal horn, especially in excitatory interneuron (Sun and Chen, 2007; Sun et al., 2009). We hypothesized that GRPR increased by EA was expressed in the spinal dorsal horn neurons.

Previous literatures indicated that 100 Hz EA promoted the release of DYN and EA at 2 Hz increased the release of beta-endorphin, endomorphin, and enkephalin. EA at 15 Hz produced a partial release of both DYNs and enkephalins (Guo et al., 1997; Ulett et al., 1998; Han, 2004). Furthermore, DYN is a critical neuromodulator of pruritus released from a specific inhibitory interneuron (Bhlhb5 neuron) in the spinal cord. Decreasing the expression of DYN in inhibitory interneurons induces pathological itch. Therefore, DYN may underlie prolonged suppression of itch by GPCRs to modulate neuronal activity. We proposed that 100 Hz EA promotes the expression and release of DYN. Consistently, previous study also showed that 100 Hz EA alleviates pruritus of atopic dermatitis-like lesions in rats induced by capsaicin injection, *via* the release of DYN (Jung et al., 2014). However, this paper does not compare the differences between high-frequency and low-frequency EA, nor does it investigate whether DYN functions by activating KOR to inhibit chronic itch and itch-specific receptor GRPR expression. Moreover, EA at 100 Hz increased the protein expression of PDYN (the precursor protein of DYN) in rats with labor pain (Jiang et al., 2016). High-frequency (100 Hz) EA enhanced the content of DYN in perfusate from the spinal cord of rats (Sun and Han, 1989) and the level of DYN-A in the plasma of heroin addicts (Mu et al., 2010) by radioimmunoassay. Here, we found that EA at 100 Hz increased the total protein expression of DYN-A but decreased the area of DYN-A immunoreactivity in the cervical spinal cord tissue. Since repeated EA promoted the release of DYN, the intracellular DYN immunoreactivity in the dorsal horn was decreased. However, western blot could detect the total content of DYN inside and outside the cells, so the expression of DYN in the tissues was up-regulated by EA. These results suggested that the 100 Hz EA promoted the expression and release of DYN-A, which is in line with the antipruritic effect of EA. Moreover, previous research has shown that KOR is one of the inhibitory GPCRs activated by DYN, an endogenous ligand of KOR (Chavkin et al., 1982). KOR agonist nalfurafine can inhibit spontaneous scratching (Inan and Cowan, 2006). Munanairi et al. found that KOR activation induced the translocation of protein kinase C (PKC)δ from the cytosol to the plasma membrane, which in turn phosphorylates and inhibits GRPR activity. A blockade of phospholipase C (PLC) prevented PKCδ translocation and GRPR phosphorylation induced by KOR agonist, suggesting that a signaling pathway of KOR–PLC–PKCδ–GRPR in the spinal cord may participate in KOR agonist-induced antipruritic effect (Munanairi et al., 2018). In this study, we found that i.p. of nor-BNI, an antagonist of KOR, significantly reversed the effect of 100 Hz EA on the inhibition of both itching behavior and GRPR expression. It suggested that the activation of KOR by DYN may participate in the effect of EA relieving itch and inhibiting GRPR expression by KOR–PLC–PKCδ–GRPR pathway.

However, Morgenweck et al. (2015) found that the nor-BNI induced acute itch in C57BL/6J mice. Kamei and Nagase have found that nor-BNI caused scratching behavior immediately after administration, which disappeared within 2 h and may be a reaction related to itching. In addition, no scratches were observed 24 h after the use of nor-BNI (Kuraishi et al., 1995). Consistently, our preliminary experiments showed that AEW mice have no more scratching behaviors after i.p. of nor-BNI, since the scratching behaviors was evaluated more than 24 h after the use of nor-BNI, and the half-life of nor-BNI in the body is about 4 h (Munro et al., 2012). Moreover, previous studies have pretreated nor-BNI about 24 h before EA to elucidate the relation between KOR and the antipruritic effect of 2 or 120 Hz EA (Han et al., 2008; Chen et al., 2013). Based on the above references and our preliminary experiments, we use nor-BNI 18–20 h before EA treatment to minimize the pruritogenic effect of nor-BNI itself. Therefore, it is very likely that nor-BNI used in our schedule acts as a selective KOR antagonist rather than a pruritogenic agent, which suggested that nor-BNI antagonized the antipruritic effect of EA.

Our study still has some limitation. We found that EA significantly improved the skin inflammation caused by AEW, so the underlying mechanism of the anti-inflammatory effect of EA other than the antipruritic effect of EA is worthy of further study.

## Conclusion

In conclusion, for the first time, we found that 100 Hz EA is the most effective frequency for relieving chronic itch, which can effectively guide the clinical treatment and improve the antipruritic effect of EA. Furthermore, we firstly investigated that EA at 100 Hz inhibits chronic itch and GRPR expression through promoting the release of DYN-A and activating KOR. In clinical observation, it was found that the effect of high-frequency EA (100 Hz) on improving chronic itch was significantly better than that of low-frequency EA (2 Hz) and antihistamine cetirizine tablets (Ni et al., 2018). Our study has better explained the neurobiological mechanism of the antipruritic effect of 100 Hz EA on chronic itch, which is helpful for the treatment of chronic pruritus in clinical practice.

## Data Availability Statement

The raw data supporting the conclusions of this article will be made available by the authors, without undue reservation.

## Ethics Statement

The animal study was reviewed and approved by the Animal Care Committee at Huazhong University of Science and Technology.

## Author Contributions

ML, X-HJ, and H-PL conceived and designed the experiments. H-PL did most of the experiments and analyzed the data. J-JL and HZ helped with the western blot experiments. CY, Z-YJ, and H-CX helped with immunofluorescence labeling experiments. L-XL, CC, and X-FH helped with the rat model and behavior test experiments. Z-EY, JC, and X-YW helped with the data collection. H-PL, X-HJ, and ML wrote the manuscript. All authors reviewed and revised this manuscript and reviewed the final version of the manuscript.

## Conflict of Interest

The authors declare that the research was conducted in the absence of any commercial or financial relationships that could be construed as a potential conflict of interest.
